# Local Polarization Unit Engineering Enables Ultrahigh Energy Density in NBT‐Based High‐Entropy Ceramic Capacitors

**DOI:** 10.1002/advs.75657

**Published:** 2026-05-19

**Authors:** Shiyu Zhou, Yucheng Zhou, Linhai Li, A. Pelaiz‐Barranco, Xuefeng Chen, Genshui Wang, Yiwei Chen, Rongjiang Wang, Konstantin Nefedev, Tengfei Hu, Dawei Wang, Tongqing Yang

**Affiliations:** ^1^ Key Laboratory of Advanced Civil Engineering Materials of the Ministry of Education Functional Materials Research Laboratory School of Materials Science and Engineering Tongji University Shanghai China; ^2^ Mechanics of Functional Materials Division Institute of Materials Science Technische Universität Darmstadt Darmstadt Germany; ^3^ The Key Lab of Inorganic Functional Materials and Devices Shanghai Institute of Ceramics Chinese Academy of Sciences Shanghai China; ^4^ Grupo de Materiales Ferroicos Facultad de Física Universidad de La Habana San Lázaro y L, Vedado La Habana Cuba; ^5^ Chengdu Weici Electronic Technology Co., Ltd Chengdu China; ^6^ Far Eastern Federal University Vladivostok Russian; ^7^ Precision Acousto‐optic Instrument Institute School of Instrumentation Science and Engineering Harbin Institute of Technology Harbin China

**Keywords:** energy storage, high‐entropy, local polar units, multilayer ceramic capacitor

## Abstract

Dielectric energy storage capacitors play a pivotal role in pulsed power systems. Herein, we demonstrate a breakthrough in dielectric energy storage by engineering ​local polarization units in high‐entropy multilayer ceramic capacitors (MLCCs). By incorporating equimolar Ba^2^^+^/Sr^2^^+^ dual cations, we precisely smoothen the phase transition and stabilize a nanoscale phase‐coexistence state in an NBT‐based matrix, which simultaneously retain robust local polar units while disrupting long‐range domain order. This unique configuration, validated by atomic‐resolution HAADF‐STEM and phase‐field simulations, enables a high reversible polarization and breakdown strength. The optimized MLCCs achieve an ultrahigh recoverable energy density of 18.2 J cm^−3^ with 91% efficiency, coupled with exceptional thermal stability and fatigue resistance. This work establishes a general design paradigm for high‐entropy dielectrics for energy storage by controlling local polarization configurations.

## Graphical abstract

Herein, we demonstrate a breakthrough in dielectric energy storage by engineering local polarization units in high‐entropy multilayer ceramic capacitors (MLCCs). The optimized MLCCs achieve an ultrahigh recoverable energy density of 18.2 J cm^−3^ with 91% efficiency, coupled with exceptional thermal stability and fatigue resistance.

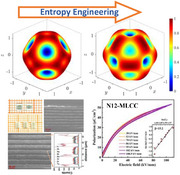

## 1 Introduction

In the fields of pulsed‐power supplies, advanced power electronics and electrified transportation, multilayer ceramic capacitors (MLCCs) have emerged as a cornerstone device platform for high‐power dielectric energy storage, owing to high power density and ultrafast charge–discharge capability, the exceptional volumetric integration enabled by thin multilayer architectures, low equivalent series resistance, and well‐established manufacturing routes [1, 2, 3, 4]. For dielectric energy‐storage MLCCs, performance is governed by the coupled electrical responses under an external applied field, spanning the recoverable energy density (*W*_rec_), energy efficiency (𝜂), breakdown strength (𝐸_𝑏_) and thermal stability. In particular, 𝑊_rec_ is tied to the polarization‐electric‐field response, 𝜂 is constrained by hysteretic and conductive losses, and 𝐸*_𝑏_* together with thermal stability defines the usable field window and the engineering safety margin [5, 6, 7, 8, 9, 10]. Therefore, the synergistic optimization of high recoverable energy density, high efficiency, and high reliability under a wide range of application conditions constitutes the key to advancing the sustainable development of dielectric energy storage materials for MLCCs.

Entropy engineering is increasingly being adopted in the research on lead‐free dielectric energy‐storage materials [11, 12, 13]. It has expanded the scope of conventional compositional design strategies and emerged as a highly active avenue for overcoming the long‐standing trade‐off between polarization, dielectric loss, and breakdown strength in dielectric systems. The core philosophy of this approach lies in the incorporation of multi‐principal components at the A‐site and/or B‐site of perovskite lattices, which elevates the configurational entropy of the material, induces pronounced chemical disorder and lattice distortion, and consequently amplifies the random internal electric fields and stresses within the matrix [14]. These structural modulations further facilitate the formation of a relaxor‐type polarization response, which macroscopically manifests as a slimmed ferroelectric hysteresis loop, reduced remanent polarization, and enhanced breakdown strength‐collectively yielding a simultaneous boost in recoverable energy density and energy efficiency. For example, Zhang and co‐workers implemented a high‐entropy design in BaTiO_3_‐based MLCCs systems to stabilize a polycrystalline relaxor phase, achieving a high discharge energy density of 20.8 J cm^−3^ while validating the feasibility of this strategy for scalable fabrication of laminated capacitor devices [3]. Concurrently, the understanding of local‐structure‐polarization response correlation in high‐entropy dielectrics is rapidly advancing: tailoring chemical short‐range order (CSRO) to steer field‐driven polarization pathways, and leveraging data‐driven or generative‐learning approaches to accelerate compositional discovery, both highlight the potential of entropy engineering to simultaneously raise breakdown strength while suppressing switching‐related dissipation [15]. Beyond room‐temperature performance, high‐entropy strategies have also been exploited to construct broader and more stable relaxor characteristics to meet practical requirements for temperature robustness [16]. In the face of challenges inherent to multicomponent complexity, a clear trend is to use controllable local disorder and random fields to weaken long‐range domain coherence while retaining robust local polar units, thereby enabling a higher fraction of reversible polarization and lower macroscopic loss [17].

Lead‐free Na_0.5_Bi_0.5_TiO_3_ (NBT)‐based relaxor ferroelectric systems combine the advantage of environmental benignity with high polarization potential [18, 19, 20, 21]. In this work, to optimize the field response without markedly compromising the strength of local polarization, we first construct a matrix (N0) by co‐substituting Ta and Zr (0.05 mol each) on the B site. The modification tunes the lattice symmetry and phase constitution (shifting the composition towards the rhombohedral‐tetragonal coexistence regime), thereby diversifying the domain configurations and reducing the coercive field while largely preserving the spontaneous polarization [22, 23]. Building on the N0, we further implement an entropy‐engineered strategy by introducing equimolar Ba^2+^ and Sr^2+^ on the A site. Exploiting the different ionic radii and local elastic fields, the dual‐cation incorporation improves A‐site size matching while enhancing compositional disorder and random‐field perturbations. Compared with single‐cation doping, the Ba^2+^/Sr^2+^ dual incorporation also smooths the phase‐transition crossover and broadens the compositional window in which multiple nanodomain states coexist, enabling a more precise regulation of polarization fluctuations and field‐induced response. With increasing Ba^2+^/Sr^2+^ content, the configurational entropy rises from 1.09R (N0) to 1.7R (N15), and the optimal enhancement of polarizability and response activation is achieved in the N12 composition. Notably, the local atomic displacements in N12 ceramics are not substantially diminished, instead, a coupled state is realized where local polarization distortion is preserved while long‐range domain correlations are disrupted, yielding a larger reversible polarization output and lower macroscopic hysteresis dissipation. Encouragingly, N12 MLCCs exhibit outstanding recoverable energy density and efficiency, together with the variations in 𝑊_rec_ and 𝜂 both less than 12% over a broad temperature range exceeding 200 °C.

## 2 Results and Discussion

**FIGURE 1:**
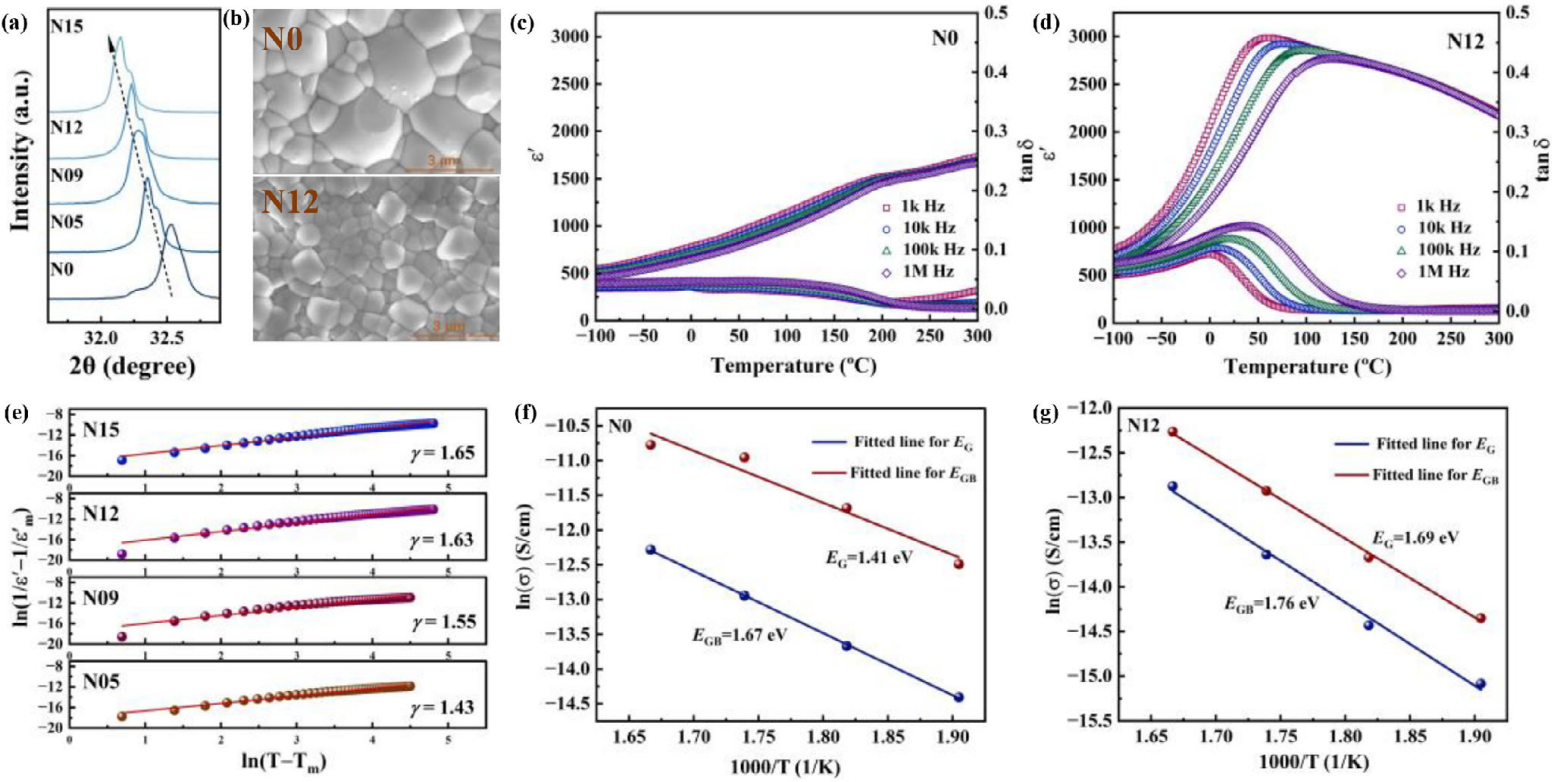
Structural and dielectric characteristics of N0‐N15 ceramics. a) Room‐temperature XRD patterns for N0‐N15 ceramics. b) SEM images of N0 and N12 ceramics. Temperature dependent *ε*′ and tan*δ* with different frequency of c) N0 and d) N12 ceramics. e) ln(1/*ε*′‐1/*ε*′_m_) versus ln(*T*‐*T*_m_) for N0‐N15 ceramics. Arrhenius fit of the grain and the grain boundary for (f) N0 and (g) N12 ceramics.

The room‐temperature polycrystalline phase structure of (Na_0.5‐_ *_x_* Bi_0.5‐_ *_x_* Ba*_x_* Sr*_x_*)(Ti_0.9_Ta_0.05_Zr_0.05_)O_3_​ ceramics is illustrated in the X‐ray diffraction (XRD) patterns (Figure S1a). The magnified view of the dominant XRD peaks centered at approximately 32.5° (Figure 1a) reveals a distinct shift toward lower angles with increasing doping content of Ba^2+/^Sr^2+^. The trend arises from the larger average cationic radius of equimolar Ba^2+^/Sr^2+^ (1.58 Å) compared with that of equimolar Na^+^/Bi^3+^ (1.35 Å), which is indicative of progressive lattice expansion [16, 24]. Figure 1b and Figure S1b–d present the scanning electron microscopy (SEM) micrographs of (Na_0.5‐_ *_x_* Bi_0.5‐_ *_x_* Ba*_x_* Sr*_x_*)(Ti_0.9_Ta_0.05_Zr_0.05_)O_3_ ceramics. All compositions exhibit a dense microstructure with well‐defined grain boundaries and no discernible porosity. With the increase of configurational entropy, the average grain size decreases gradually from 1.13 µm for the N0 ceramic to 0.59 µm for the N15 counterpart, which suggests that the increased compositional disorder within the lattice suppresses the driving force for grain growth and elevates the grain boundary density per unit area, thereby facilitating the enhancement of *E*_b_ [16, 25]. The statistical histograms of average grain sizes for the five ceramic compositions are provided in Figure S2a–e.

Figure 1c,d and Figure S3a–c show the temperature‐dependent relative permittivity (*ε*′) and loss factor (tan*δ*), for (Na_0.5‐_ *_x_* Bi_0.5‐_ *_x_* Ba*_x_* Sr*_x_*)(Ti_0.9_Ta_0.05_Zr_0.05_)O_3_​ ceramics. All doped samples exhibit frequency dispersion along with broadened dielectric peaks, and the characteristic permittivity peak progressively shifts towards room temperature as the doping content increases. The behavior stems from the incorporation of larger‐radius Ba^2+^/Sr^2+^ cations, which (i) drives the phase stability towards higher‐symmetry tetragonal/cubic states and (ii) enhances local random‐field perturbations, thereby strengthening relaxor characteristics [26]. Such inference is further corroborated by the gradually increasing slope (*γ*) derived from the Curie‐Weiss law fitting (Figure 1e) [4]. In addition, the *ε′*_m_ at the temperature‐corresponding dielectric peak (*T*_m_) initially increases from N0 to N12 and then decreases for N15, which indicates that compositions from N05 to N12 progressively approach a multiphase‐coexistence boundary, leading to lattice softening and an increased population of polarizable units that contribute to the dielectric response. By contrast, for N15, the downturn *ε′*_m_ in is attributed to the synergistic effects of excessively strong random fields caused by severe cation disorder and the over‐dilution of Bi‐based polarization sources. Complex impedance spectra recorded from 525°C to 600°C for N0 and N12 ceramics, together with the frequency dependence of *M″* and *Z″* at 575 °C are presented in Figure S4a,c and b,d, respectively. Compared with N0, N12 exhibits a higher resistivity and enhanced electrical homogeneity [27]. Arrhenius plots derived from the impedance data and fits using a two‐series RC equivalent‐circuit model is shown in Figure 1f,g. Both the grain‐boundary and grain conduction activation energies (*E*_GB_ and *E*_G_) increase for N12 and become more comparable, providing direct evidence for enhanced electrical insulation and thus ensuring the high *E*_b_ in N12 ceramics [16, 28].

**FIGURE 2:**
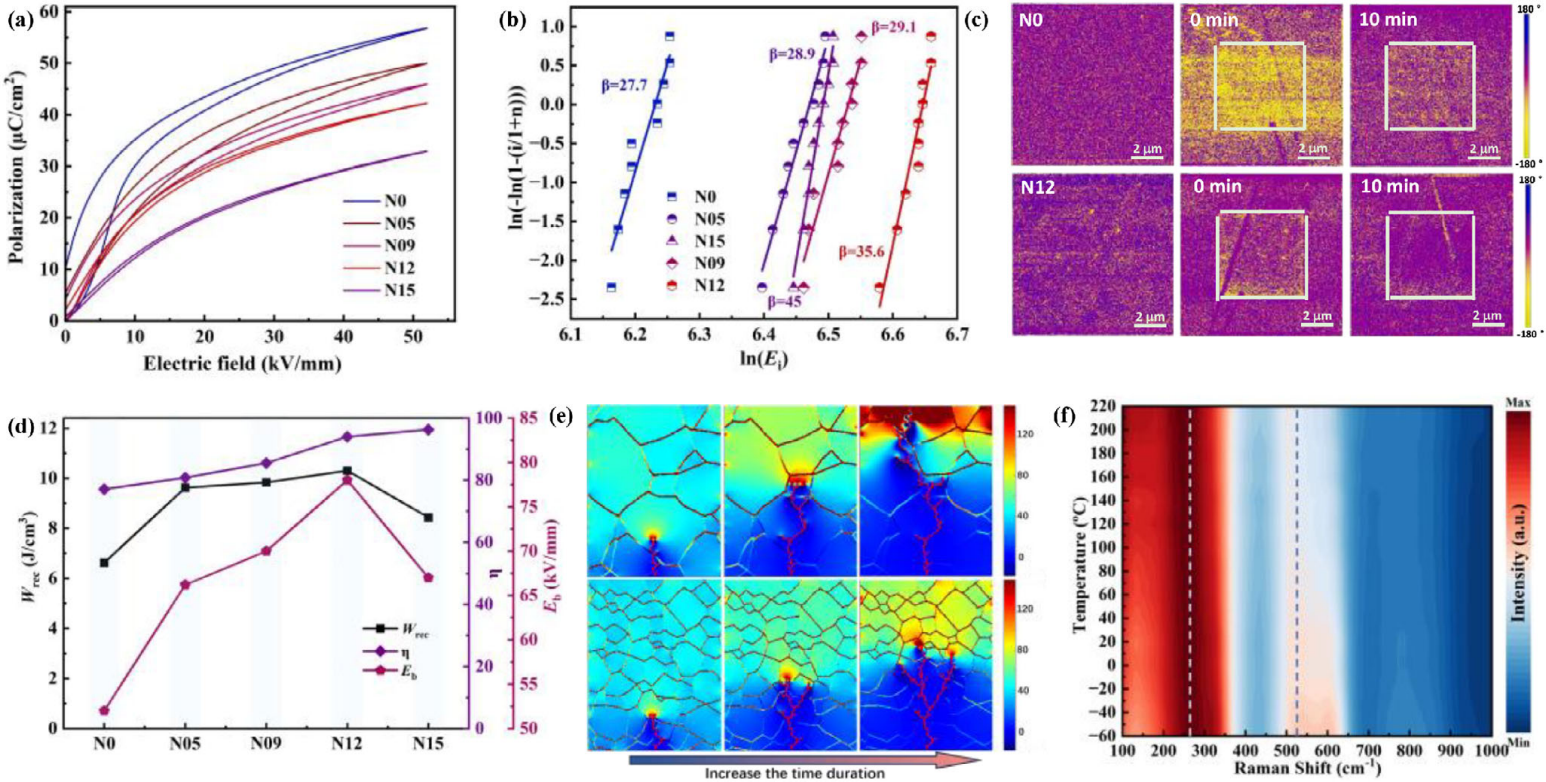
Electrical characterization of the N0–N15 ceramics and assessment of the optimum‐performing composition. a) Unipolar *P*–*E* loops of N0‐N15 ceramics under 50 kV/mm at 10 Hz. b) Weibull distribution of the N0‐N15 ceramics. c) Out‐of‐plane PFM phase images before and after poling treatment with 10 min. d) *W*_rec_, *η* and *E*_b_ of the N0‐N15 ceramics. e) Simulation of breakdown path distribution of N0 and N12 ceramics. (f) Raman spectra of N12 ceramics from ‐60°C to 220°C.

Figure 2a presents the unipolar *P*–*E* hysteresis loops of N0‐N15 ceramics measured under an electric field of 50 kV mm^−1^. As configurational entropy increases, the long‐range domain correlations are progressively disrupted, rendering polarization more reversible and leading to a gradual reduction in the remnant polarization (*P*_r_). Owing to the dilution of Bi^3+^ (a dominant polarization source) and the disruption of long‐range order, the maximum polarization (*P*_max_) of N05‐N12 ceramics decreases slightly [6]. Notably, the polarization of N05 ceramic at the *E*_b_ is comparable to that of N0, which can be attributed to the more facile establishment of field‐induced polarization (Figure S5a). In contrast, for N15 ceramic, the further increase in internal grain disorder restricts the effective polarization that can be “activated” by the external electric field, resulting in a significant reduction in *P*_max_. The reliability of the measured *E*_b_ for all compositions was verified using Weibull statistics (Figure 2b), yielding shape parameters *β* > 20 [3]. The energy‐storage performance at the respective *E*_b_ was then evaluated for the five compositions (Figure 2d). Considering both *W*_rec_ and *η*, the N12 ceramic delivers outstanding room‐temperature performance, with *W*_rec_ ≈ 10.5 J cm^−3^ and *η* ≈ 94.5%. The unipolar *P*–*E* hysteresis loops and the statistical analysis of energy storage performance under varying electric fields for N12 ceramic are provided in Figure S5b,c.

Piezoelectric force microscopy (PFM) measurements (Figure 2c) track the evolution of polarization response in N0 and N12 ceramics under external electric fields. The initial phase images show that neither sample exhibits long‐range ordered macroscopic ferroelectric domains. A domain “writing” process was performed on a 5 × 5 µm central area of both samples. Distinct domain signals were detected in the N0 ceramic after “reading,” with partial domains remaining unswitched even after 10 min. In contrast, little domain signals were observed in the N12 ceramic. It indicates that the increase in configurational entropy enhances local elastic fluctuations, which further fragments the internal domain structure of N12 ceramic and reduces domain size‐an effect conducive to mitigating hysteresis and improving energy storage efficiency [29, 30]. On the other hand, finite element analysis (FEA) was employed to simulate the local electric field distribution based on the microstructural and dielectric characteristics of N0 and N12 ceramics. The elevated grain boundary density significantly enhances breakdown resistance and suppresses the formation of localized high electric fields [31, 32]. Consequently, the electrical treeing propagation length in N12 ceramic is only approximately 55% of that in N0 ceramic at the breakdown field of the latter, verifying the improved breakdown endurance of N12 ceramic. In addition, the temperature‐dependent Raman spectra of the N12 ceramic (Figure 2f) and the magnified views (Figure S5d–f) demonstrate that the B‐site‐related mode near ≈300 cm^−1^ remains diffuse and shifts only slightly towards lower wavenumbers upon heating. Meanwhile, the doublet peaks of the BO_6​_ octahedra at approximately 500 cm^−1^ maintain an intensity trend of higher left peak and lower right peak from ‐40°C to 165°C, with the intensity difference between the two peaks gradually increasing. The features indicate that N12 ceramic evolves from a state with strong local order at low temperatures to a relaxor state characterized by diffuse phase transition and phase coexistence, which is favorable for maintaining excellent energy storage temperature stability across a broad temperature range [33, 34]. Collectively, the above results underscore the promise of N12 composition for energy‐storage capacitor applications.

**FIGURE 3:**
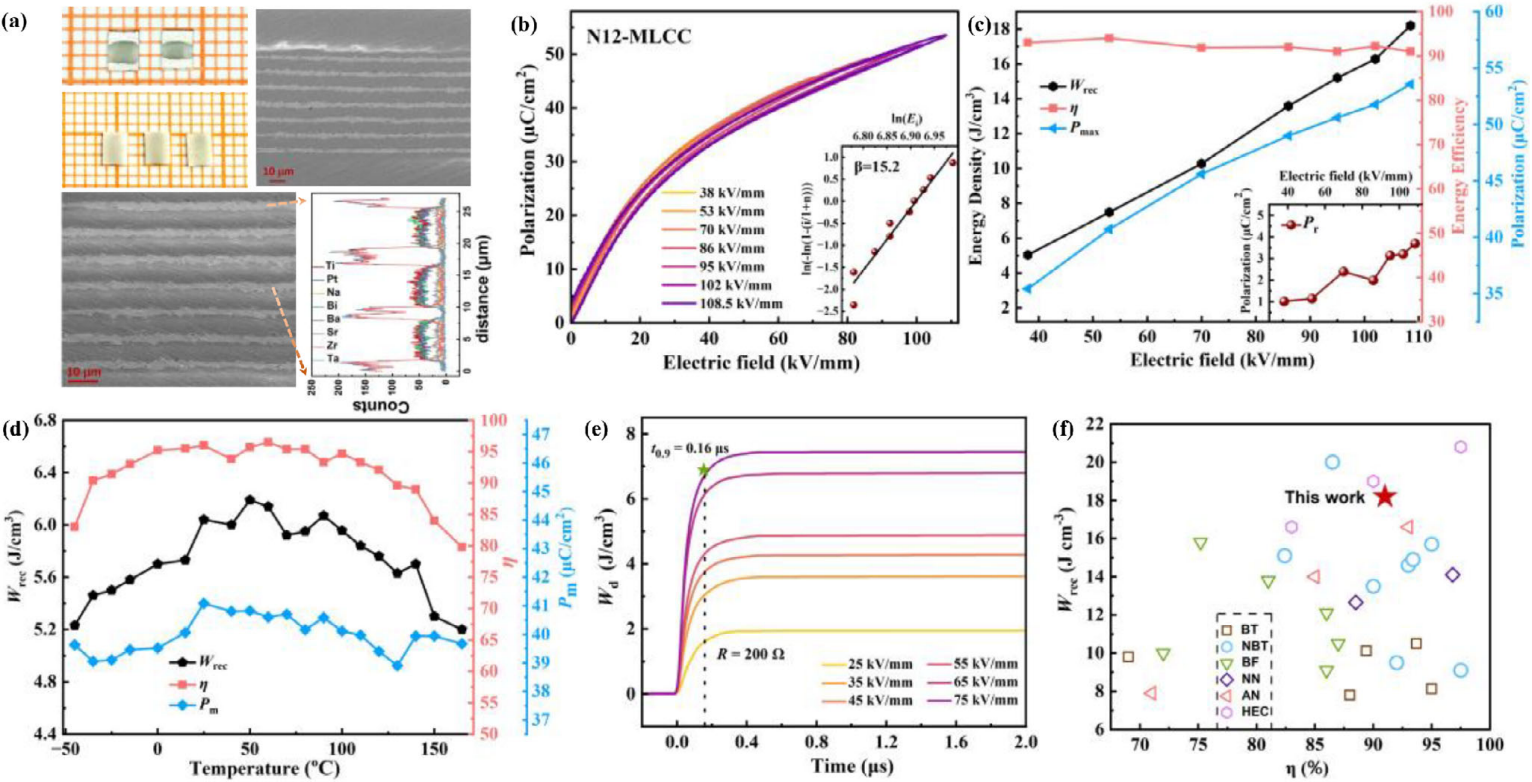
Energy storage performance of the N12 MLCCs. a) Macroscopic and Microscopic Morphology of N12 MLCCs. b) Unipolar *P*–*E* loops c) *W*_rec_, *P*_max_, *η* and *P*_r_ of the N12 MLCCs with different electric fields at 10 Hz. d) *W*_rec_, *P*_m_ and *η* of the N12 MLCCs from ‐45°C to 165°C under the 40 kV mm^−1^ at 10 Hz. e) Discharge energy density‐time curves of the N12 MLCCs. f) Comparation of *W*_rec_ and *η* between N12 MLCCs and other reported lead‐free MLCCs.

Figure 3a summarizes the device architecture of the N12‐based MLCC. Macroscopically, each sintered MLCCs possess a footprint of 2 cm × 3 cm. Microstructurally, the N12 MLCCs comprise six effective dielectric layers with uniform thickness uniformity, and each dielectric layer is about 7 µm thick. The electrode layers are around 2–3 µm thick and remain dense, continuous and free of noticeable discontinuities. The linear sweep energy‐dispersive X‐ray spectroscopy (EDS) profile across three dielectric layers clearly reveals the elemental distribution discrepancy between dielectric and electrode layers, with homogeneous elemental distribution in each layer and no obvious diffusion or aggregation phenomena observed. Figure 3b presents the *P*–*E* hysteresis loops and Weibull statistical analysis plots of the N12 MLCCs under varying electric fields. The high Weibull modulus attests to the excellent structural uniformity of the fabricated devices [35, 36]. As the breakdown electric field is further increased to 108.5 kV mm^−1^, the *P*_m_ of the N12 composition is further enhanced to 53.5 µC cm^−2^, while maintaining a low hysteresis level (*P*_r_​ < 3.6 µC cm^−2^). Consequently, the N12 MLCCs achieve an ultrahigh *W*_rec_​ of up to 18.2 J cm^−3^ with an excellent *η* of 91%, as shown in Figure 3c. In addition, temperature‐dependent and cycling fatigue tests (Figure S6a,b) demonstrate that the N12 MLCCs exhibit excellent wide‐temperature stability (‐45°C–220°C, Δ*W*_rec_ ​< 12%, Δ*η* < 12%) and superior cycling reliability (4×10^6^ cycles, Δ*W*_rec_​ < 5.4%, Δ*η* < 4%), with the corresponding statistical data summarized in Figure 3d and Figure S6c. Finally, charge‐discharge performance evaluations (Figure 3e and Figure S7a,b) indicate that the N12 MLCCs deliver a high discharge energy density (*W*_d_​ ≈ 7.5 J cm^−3^) and a fast discharge speed (*t*_0.9_ ​ ≈ 160 ns) under a load resistance of 200 Ω and an electric field of 75 kV mm^−1^. Compared with other lead‐free MLCCs reported in recent years (Figure 3f), the N12 MLCCs exhibit outstanding comprehensive energy storage performance, rendering it a highly promising candidate material for next‐generation high‐power pulsed discharge capacitor technologies [3, 4, 6, 18, 19, 20, 24, 25, 26, 27, 28, 29, 32, 35, 36, 37, 38, 39, 40, 41, 42, 43, 44, 45, 46, 47, 48, 49].

**FIGURE 4:**
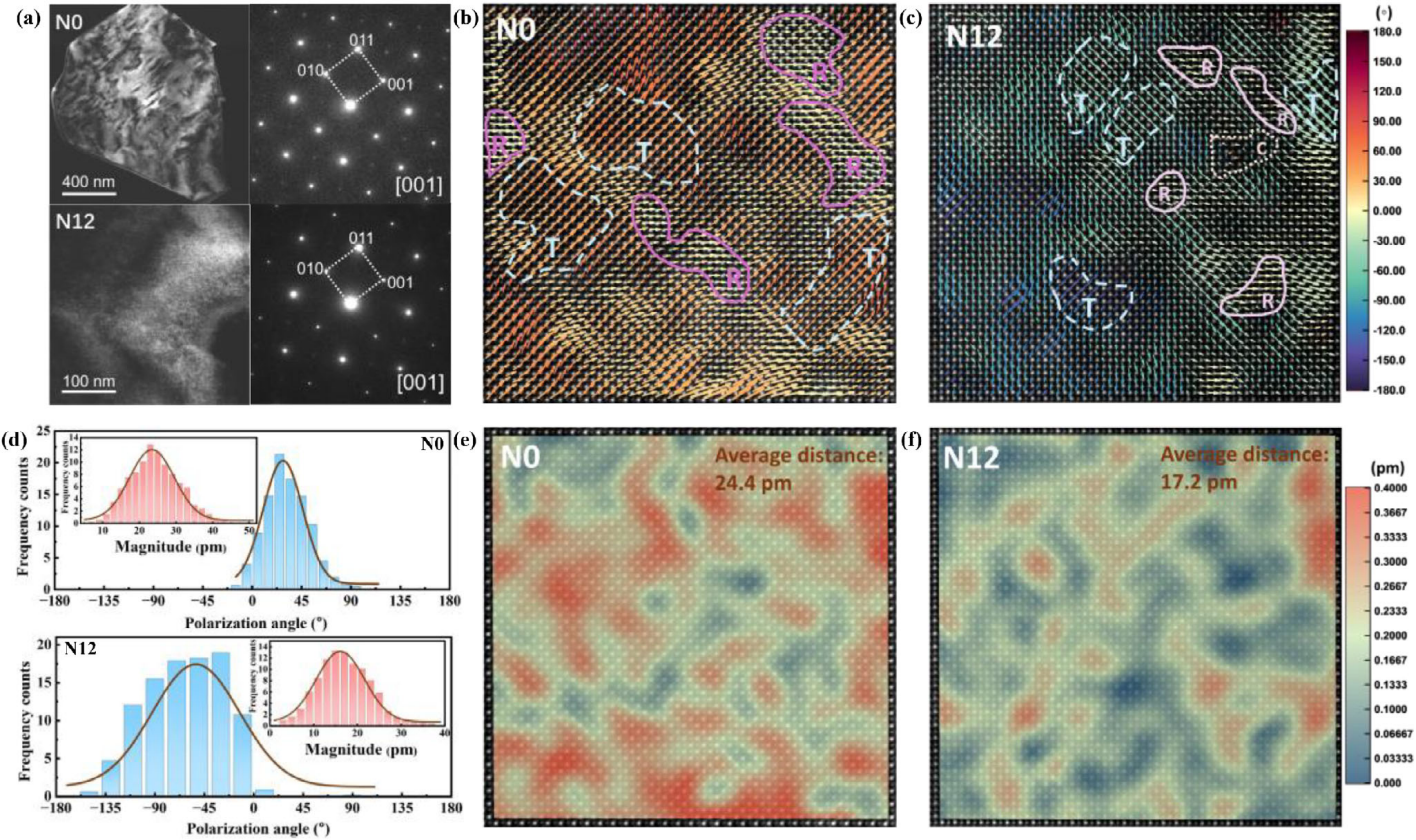
Atomic‐scale structural analysis of N12 ceramics along [001]_c_. a) Morphology and SAED patterns of the N0 and N12 ceramics. HAADF‐STEM polarization vector images of the b) N0 and c) N12 ceramics. d) Statistic of calculated polarization angle and polarization magnitude distribution of the N0 and N12 ceramics. HAADF‐STEM polarization magnitude images of the e) N0 and f) N12 ceramics.

To gain mechanistic insight into the evolution of polarization configurations and their implications for energy‐storage behavior, high‐resolution transmission electron microscopy (HRTEM) and high‐angle annular dark‐field (HAADF) STEM characterizations were performed for the N0 and N12 ceramics along the [001]_c_ zone axis, with the original images shown in Figure 4a and Figure S8a,b, respectively. The SAED patterns of the samples show a near‐cubic phase (*Pm*‐3*m*) symmetry and no other diffraction spots are present. The HRTEM micrographs reveal that the N0 ceramic exhibits irregular lamellar/stripe‐like domains within the grains spanning submicrometric to nanometer length scales, indicating that long‐range domain order has already been partially disrupted [6, 19]. By contrast, N12 ceramic exhibits a highly diffuse contrast with domain features fully confined to the nanometer scale, consistent with a relaxor state characterized by short‐range polar order. Further analysis of the polarization angles of off‐center atoms derived from 2D Gaussian fitting (Figure 4b,c and Figure S8c,d) demonstrate that the N0 ceramic possesses distinct rhombohedral (R) and tetragonal (T) phase polar units with characteristic sizes of ≈5–7 nm. In N12 ceramics, the polar units are further refined to ≈2–3 nm, and the R/T distribution becomes more disordered, accompanied by a small fraction of nearly displacement‐free cubic phase‐like (C) regions. The corresponding polarization‐angle statistics are summarized in Figure 4d. By classifying displacements along [110] and equivalent directions as R‐like, along [010] and equivalent directions as T‐like, and regions with displacement magnitudes <8 pm as C‐like, the phase fractions are quantified as follows: 59% R phase and 41% T phase for N0 ceramic, versus 42% R phase, 53% T phase and 5% C phase for N12 ceramic [16, 50, 51]. These results indicate that increasing configurational entropy and atomic‐scale chemical disorder drives N12 towards a phase‐coexistence boundary while retaining a large population of polar units that can readily support field‐induced polarization. Analysis of the off‐centered displacement vectors (Figure 4e,f) further shows that the mean displacement amplitude in N12 ceramic (17.2 pm) is still considerable compared with that in N0 ceramic (24.4 pm), rather than being severely diminished [19, 20]. The observation verifies that the entropy engineering strategy does not achieve relaxor behavior at the expense of excessively compromising polarization; instead, it realizes a unique state in which robust local polar units persist while long‐range order is disrupted. Atomic‐resolution EDS mapping (Figure S9) reveals the absence of cationic periodic ordering or cluster aggregation, confirming the homogeneous distribution of the seven cations in the matrix. Such a configuration provides a microscopic basis for enlarging the reversible polarization contribution, reducing hysteretic losses and ultimately enabling superior energy‐storage performance.

**FIGURE 5:**
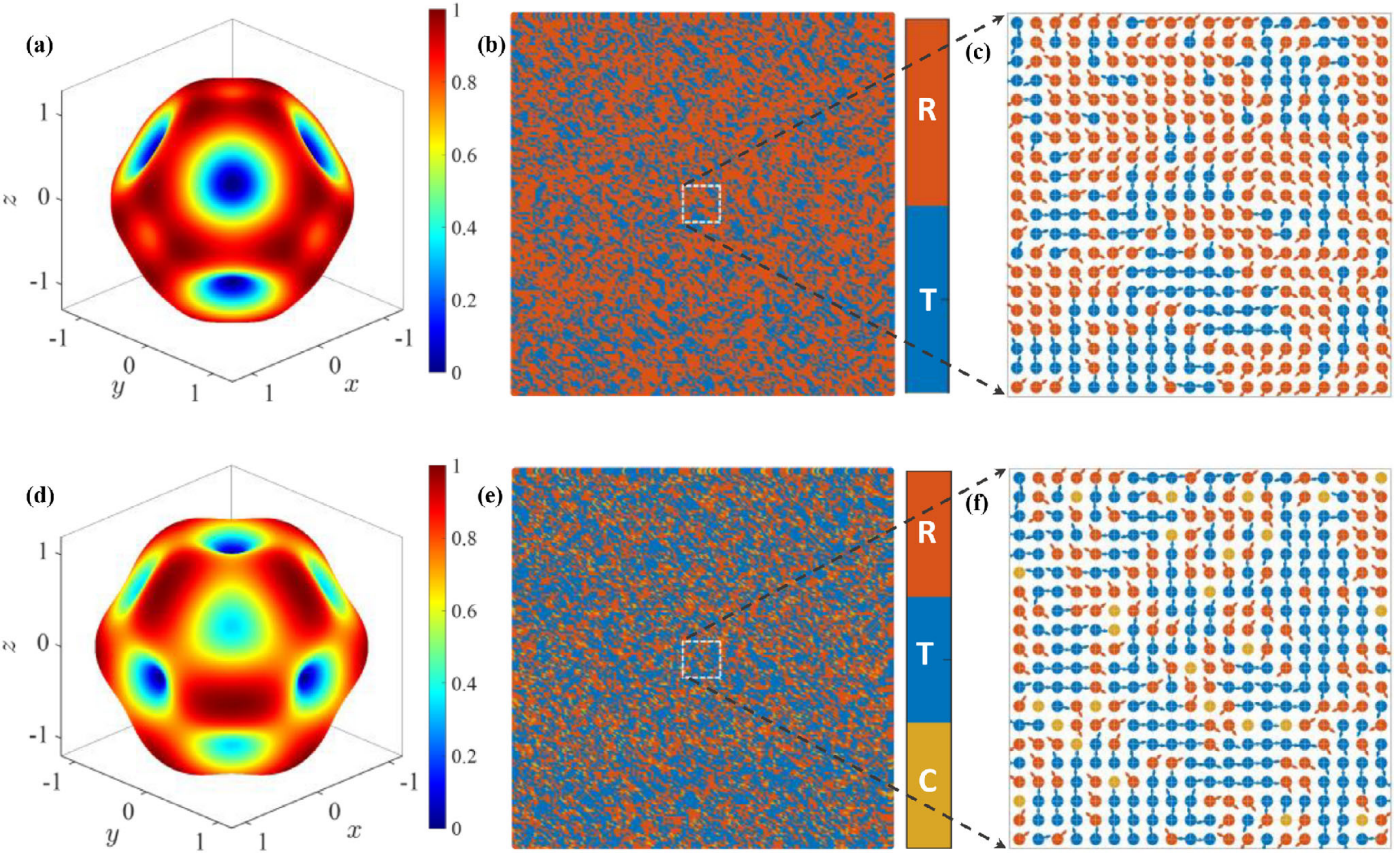
Phase‐field simulations for N0 and N12 ceramics. Landau free energy landscapes of the a) N0 and d) N12 ceramics. Simulated 2D phase distribution maps and enlarged views for b, c) N0 and e, f) N12 ceramics.

Finally, phase‐field simulations were employed to provide further insights into the phase transition behavior and polarization origin of the N0 and N12 high‐entropy ceramics. Figure 5a,b shows the Landau free‐energy landscapes constructed for N0 and N12 samples. In thermodynamics, a lower Landau free energy value corresponds to a higher phase stability in the relevant orientation: the global energy minima along the diagonal direction correspond to the R phase, while those along the coordinate axes correspond to the T phase [18, 52]. The presence of energy minima in both orientations confirms the coexistence of R and T phases in both compositions. However, compared with the N0 composition, the free energy barriers of the N12 sample exhibits a much smoother transition, indicative of enhanced relaxor characteristics [53, 54]. Meanwhile, the free energy depths of the two phases become more comparable, with the T phase possessing a lower energy state, suggesting that the T phase is more thermodynamically favorable and thus accounts for a larger fraction. Figure 5b,e presents the simulated 2D phase distribution maps of the N0 and N12 ceramics. It is evident that the R phase remains dominant in the N0 sample, whereas the N12 sample exhibits a near‐equal ratio of R and T phases accompanied by scattered C phase regions, which arises from the elevated local chemical disorder. The magnified views of the central regions (Figure 5c,f) enable a clearer visualization of the polarization orientations at the nodes: the R and T phases each exhibit four distinct polarization directions and the C phase shows negligible polarization displacement. It can be observed that, relative to N0 composition, the N12 ceramics displays a modest reduction in polar‐unit size together with a more disordered distribution, in close agreement with the HAADF‐STEM observations. The calculated P‐E hysteresis loops of the two samples' domain structures at room temperatures are depicted in Figure S10a, b, which are highly consistent with the testing data. Collectively, the simulation results are in excellent agreement with the HAADF‐STEM characterization, which further validates the feasibility and effectiveness of the entropy engineering strategy for tailoring local polar units and thus enhancing energy storage performance of high‐entropy ceramics.

## 3 Conclusion

In summary, we establish an entropy engineering design framework that tailors local polar units of an NBT‐based matrix to deliver an exceptional balance of dielectric energy storage metrics. The incorporation of equimolar Ba^2+^/Sr^2+^ introduces intense random field fluctuations and smoothes the phase transition behavior, enabling the precise identification of the N12 composition with the optimal polarization response. At the microscale, the N12 high‐entropy ceramic retains robust local polar units while maintaining a multi‐phase coexistent state with high atomic‐scale chemical disorder. Furthermore, the high internal disorder suppresses grain boundary migration and enhances electrical resistivity. At the macroscale, the N12 ceramic exhibits low hysteresis loss, high saturated polarization, and elevated breakdown strength. Ultimately, the N12‐based MLCCs exhibit an outstanding *W*_rec_ of 18.2 J cm^−3^ with a high *η* of 91%. This work highlights the design flexibility and great potential of high‐entropy ceramics for dielectric energy storage applications, and provides a universally applicable design paradigm for developing high‐performance dielectric energy storage capacitors for practical applications.

## Conflicts of Interest

The authors declare no conflicts of interest.

## Supporting information




**Supporting File**: advs75657‐sup‐0001‐SuppMat.docx.

## Data Availability

The data that support the findings of this study are available from the corresponding author upon reasonable request.
