# Supplementary material for: Local Polarization Unit Engineering Enables Ultrahigh Energy Density in NBT‐Based High‐Entropy Ceramic Capacitors
Source: Adv Sci (Weinh). 2026 May 19;13(44):e75657. doi: 10.1002/advs.75657 (PMC13335951; doi:10.1002/advs.75657)
Supplement: Supplementary file 1 [file ADVS-13-e75657-s001.docx]

Supporting Information

**Local Polarization Unit Engineering Enables Ultrahigh Energy Density in NBT-based High-Entropy Ceramic Capacitors**

*Shiyu Zhou, Yucheng Zhou^*^, Linhai Li, A. Pelaiz-Barranco, Xuefeng Chen, Genshui Wang ^c^, Yiwei Chen, Rongjiang Wang, Konstantin Nefedev, Tengfei Hu^*^, Dawei Wang^*^, Tongqing Yang^*^*

**Materials and Methods**

**1. Sample Preparation**

(Na_0.5-_*_x_*Bi_0.5-_*_x_*Ba*_x_*Sr*_x_*)(Ti_0.9_Ta_0.05_Zr_0.05_)O_3_ (abbreviated as N0 for *x*=0, N05 for *x*=0.05, N09 for *x*=0.09 N12 for *x*=0.12 N15 for *x*=0.15) ceramics were fabricated via a conventional solid-state reaction method with high purity raw chemicals of Na_2_CO_3_ (≥99.8%), Bi_2_O_3_ (≥99.9%), TiO_2_ (≥99%), Ba_2_CO_3_ (≥99.95%), Sr_2_CO_3_ (≥99.99%), ZrO_2_ (≥99.99%), Ta_2_O_5_ (≥99.99%). All the chemicals were dried at 200 ºC, weighted according to the stoichiometric amounts and then milled for 18 hours in the zirconia jars with ethanol and zirconia balls. The well-mixed powders were dried and calcined at 850 ºC for 4 hours and then ball milled again for 12 hours. Subsequently, the dried powders were mixed with butanone, glycerol trioleate, dibutyl phthalate, polyvinyl butyral, and polyethylene glycol and ball milled for 12 hours to obtain a uniform ceramic slurry. The ceramic green sheets were fabricated by using a tape-casting method. After drying and cutting, the square tapes were stacked and hot-pressed at 65 ºC. Subsequently, four layers of the green tapes were stacked and laminated using a parallel-plate hot press under a pressure of 20 MPa. After sintering, the resulting ceramic samples achieved a final thickness of about 50 μm. Finally, the green ceramic samples were maintained at 480 ºC for 8 hours to remove the organics and sintered at 1100-1200 º C for 2-3 hours in air. The N12 dry powder was used to fabricate multi-layer ceramic capacitors (MLCCs) using a roll-to-roll tape casting machine, in which the slurry reservoir remained fixed while the PET film moved continuously. This configuration is better suited for producing longer ceramic tape lengths for subsequent MLCC fabrication. In addition, the system integrates an in-line drying function during tape transport, allowing the as-cast continuous tape to proceed directly to the next processing step. A high temperature platinum slurry (composed of pure Pt particle and ZrO_2_ containing glass powder) was printed employing a screen printer to prepare internal electrodes, followed by stacking and aligning via hot isostatic lamination machine. Same procedure was used for removing organic medias and sintering as for the ceramic green sample mentioned above. The obtained MLCCs were polished and washed with alcohol and then coated with silver electrodes.

**2. Structure Characterization**

All samples were examined by XRD (Cu-Kα source) on a Bruker D8 Advance [diffractometer](https://www.sciencedirect.com/topics/earth-and-planetary-sciences/diffractometers). [XRD patterns](https://www.sciencedirect.com/topics/earth-and-planetary-sciences/diffraction-pattern) were collected over a range of 2θ = 20° - 70°, with 0.02° per scanning step. The grain morphologies and element distribution of the sintered ceramic samples and MLCCs were detected by a field-emission scanning electron microscope (FE-SEM, Quanta 200FEG, FEI Company, USA). The Raman spectra were collected using a Raman scattering spectrometer (LabRAM HR Evolution, Horiba, France) with a heating stage (Linkam, THM600, UK) at 532 nm laser excitation from -60 ºC to 220 ºC. A piezo-response force microscope (PFM, Dimension Icon, Bruker, Germany) equipped with a bias voltage-adding device was adopted to detect the surface morphology and the phase angle distribution. The PFM sample was well polished to a roughness of several nanometer and the scanning aera is 5 μm × 5 μm. The ceramics were prepared as a TEM thin-foil sample using traditional steps, including cutting, grinding, polishing, ion-milling and coating thin carbon films. A JEM-F200 microscope was used to acquire dark-field images and selected area electron diffraction patterns. The atomic-scale high-angle annular dark-field (HAADF) imaging was carried out on a Cs-corrected Spectra300 microscope with a convergence/collection semi-angle of 30mrad/50‒200mrad. The polarization vectors were determined by one B-site cation relative to the center of four A-site cations. The atomic column positions at picometer-precision fitting were performed using MATLAB software.

**3. Electric Property Measurements**

The *P*-*E* loops measured at room temperature were derived from the ferroelectric measurement system (Radiant Technologies Inc., Albuquerque, USA). For the test of temperature-dependent P-E loops, the ferroelectric measurement system (TF analyzer 2000, Aachen, Germany) was used with a low-temperature probing stage (Janis, USA) and a high-temperature probing stage (TFA 370-7, aixACCT, Germany). Dielectric charge-discharge measurement system (CFD-003, Tongguo Technology, China) was used to measure the energy release properties. The storage energy was discharged under a load resistance R_L_ of 200 Ω. The electrode size of ceramic samples used for the two energy-related measurements is ~1.0-2.0 mm in diameter and ~0.05-0.10 mm in thickness, and the electrode size of the MLCCs sample is ~7 μm (thickness) × 3.93 mm^2^. For dielectric properties and impedance measurements, the ceramics were polished into a thickness of ~0.9 mm with a diameter of ~4 mm. The electrodes were deposited by magnetron sputtering at a current of 10 mA for 5 minutes. The electrodes were deposited by magnetron sputtering at a current of 10 mA for 5 minutes. Dielectric and impedance measurements are performed using a precision LCR meter (E4980A, Agilent Technologies, USA) combined with a temperature control system (DMS-2000, Balab Technologies, China). The heating rate was set at 3 °C min^-1^, and measurements were performed within a test frequency range of 1 kHz to 1 MHz.

**4. Phase field model**

The phase-field model utilized in this work describes the coupled electromechanical behavior of dislocations in ferroelectric materials through a total free energy functional^[1-3]^. The total free energy functional $F$ is defined as an integral over the volume $V$ of the material, encompassing contributions from the bulk Landau-type energy, electrostatic energy, polarization gradient energy, elastic energy, and random field energy:

$$F=\int_{V} \left[ f_{\text{bulk}}(\mathbf{P})+f_{\text{elec}}(\mathbf{P},\mathbf{E})+f_{\text{grad}}(\nabla\mathbf{P})+f_{\text{elas}}(\boldsymbol{\epsilon},\mathbf{P})+f_{\text{rand}} \right]\text{ }dV.$$

The bulk free energy density $f_{\text{bulk}}$ is expressed as a high-order polynomial in the polarization components $P_{i}$ using composition-dependent Landau–Devonshire coefficients,

$f_{\text{bulk}}=a_{ij}P_{i}P_{j}+a_{ijkl}P_{i}P_{j}P_{k}P_{l}+a_{ijklmn}P_{i}P_{j}P_{k}P_{l}P_{m}P_{n}$,

where $a_{ij}$, $a_{ijkl}$, and $a_{ijklmn}$ are the Landau-Devonshire potential coefficients. Specifically,

$$f_{\text{bulk}}= a_{1}(P_{1}^{2}+P_{2}^{2}+P_{3}^{2})+a_{11}\left( P_{1}^{4}+P_{2}^{4}+P_{3}^{4} \right)+a_{12}\left( P_{1}^{2}P_{2}^{2}+P_{2}^{2}P_{3}^{2}+P_{3}^{2}P_{1}^{2} \right)$$

$+a_{111}\left( P_{1}^{6}+P_{2}^{6}+P_{3}^{6} \right)+a_{112}\left[ P_{1}^{2}\left( P_{2}^{4}+P_{3}^{4} \right)+P_{2}^{2}\left( P_{1}^{4}+P_{3}^{4} \right)+P_{3}^{2}\left( P_{1}^{4}+P_{2}^{4} \right) \right]+a_{123}P_{1}^{2}P_{2}^{2}P_{3}^{2}$.

The electrostatic energy density is given by $f_{\text{elec}}=-\frac{1}{2}K_{ij}E_{i}E_{j}-P_{i}E_{i}$, where $K_{ij}=\varepsilon_{0}\kappa\delta_{ij}$ represents the dielectric tensor, with $\varepsilon_{0}$ being the vacuum permittivity and $\kappa$ the relative background permittivity. The electric field derives from the electric potential as $E_{i}=-\phi_{,i}$. The gradient energy density takes the isotropic form $f_{\text{grad}}=\frac{1}{2}g_{ijkl}P_{i,j}P_{k,l}$. The elastic energy density incorporates both polarization-induced eigenstrain and the eigenstrain field of dislocations:

$$f_{\text{elas}}=\frac{1}{2}c_{ijkl}(\epsilon_{ij}-\epsilon_{ij}^{p})(\epsilon_{kl}-\epsilon_{kl}^{p}),$$

where $\epsilon_{ij}=(u_{i,j}+u_{j,i})/2$ is the total strain, $\epsilon_{ij}^{p}=Q_{ijkl}P_{k}P_{l}$ is the electrostrictive eigenstrain. Higher-order electrostriction is neglected, as the associated lattice spacing changes remain well below the threshold for nonlinear effects. The random field energy is given by $f_{\text{rand}}=-E_{i}^{\text{rand}}P_{i}$, and $E_{i}^{\text{rand}}$ denotes the random field arising from chemical inhomogeneity follows a Gaussian distribution $N$, $E_{i}^{\text{rand}}\sim N\left( \mu, \Delta\right),$where its expectation $\mu$ is set to be zero in the simulation, and the standard deviation is denoted as $\Delta$.

From this energy functional, the constitutive relations for stress and electric displacement are derived via thermodynamic differentiation:

$$\sigma_{ij}=\frac{\partial f}{\partial\epsilon_{ij}}=c_{ijkl}(\epsilon_{kl}-\epsilon_{kl}^{p}), D_{i}=-\frac{\partial f}{\partial E_{i}}=K_{ij}E_{j}+P_{i}.$$

The governing field equations consist of the mechanical equilibrium equation, Gauss’s law for dielectrics, and a time-dependent Ginzburg–Landau equation describing polarization evolution:

$$\sigma_{ij,j}=0, D_{i,i}=0, \frac{\partial P_{i}}{\partial t}=-M\frac{\delta F}{\delta P_{i}},$$

where $M$ is a kinetic mobility coefficient.

The coupled system of governing equations is discretized and solved numerically using the finite element method. The simulation domain is a two-dimensional region measuring 100 nm × 100 nm, discretized with a uniform quadrilateral mesh of size 0.8 nm × 0.8 nm. Mechanically, stress-free boundary conditions are applied on all external surfaces, i.e.,

$$\sigma_{ij}n_{j}=0 \text{on }\partial V.$$

Electrically, a zero Neumann condition is imposed on the electric displacement normal to the boundary,

$$D_{i}n_{i}=0 \text{on }\partial V,$$

which corresponds to a zero surface charge condition. The polarization evolution follows the time-dependent Ginzburg–Landau equation with a natural boundary condition of zero polarization flux at the surfaces. The bulk free energy is described by a composition-dependent Landau–Devonshire potential, where the coefficients vary spatially according to the local material composition. The complete set of Landau potential parameters $a_{1}-a_{123}$ for N0 and N12 used in the simulations are listed in Table 1.

Table 1: Coefficients of Landau-Devonshire potential for N0 and N12

| Coefficients | N0 | N12 | Units |
| --- | --- | --- | --- |
| a_1_ | -124.66 | -120.80 | aC^−2^ nm^2^ pN |
| a_11_ | 60 | -150 | aC^−4^ nm^6^ pN |
| a_12_ | -4210 | -4175 | aC^−4^ nm^6^ pN |
| a_111_ | 5000 | 5000 | aC^−6^ nm^10^ pN |
| a_112_ | 74370 | 74370 | aC^−6^ nm^10^ pN |
| a_123_ | 123210 | 123210 | aC^−6^ nm^10^ pN |

**Figure S1.** a) XRD patterns of N0-N15 ceramics. b-d) SEM images of N05, N09 and N15 ceramics.

**Figure S2.** a-e) Average grain size diagrams of the N0-N15 ceramics.

**Figure S3.** a-c) Temperature dependent ε′ and tanδ with different frequency for N05, N09 and N15 ceramics.

**Figure S4.** Complex impedance at 525-600 ºC and Z′′ and M′′ plots at 575 ºC for a, c) N0 and b, d) N12 ceramics.

**Figure S5.** a) Unipolar *P*-*E* loops prior to the breakdown electric fields of the N0-N15 ceramics. b) Unipolar P-E loops, c) *W*_rec_, *P*_max_, *η* and *P*_r_ and d-f) Raman spectra from -60 ºC to 220 ºC of the N12 ceramics. Among them, Figure S5 d, e) are the magnified Raman spectra curves at ~300 cm^-1^ and 510 cm^-1^ presented by 3D contour map and Figure S5 f) is the 2D Raman spectra.

**Figure S6.** a) Unipolar P-E loops from -85 ºC to 240 ºC and b) fatigue test Unipolar P-E loops of the N12 MLCCs under the 40 kV/mm. c) Statistical plots of *W*_rec_, *W*_st_, and *η* for N12 MLCCs.

**Figure S7.** a) Discharge current-time curves *P-E* loops and b) *W*_d_ and *t*_0.9_ of the N12 MLCCs.

**Figure S8.** a, b) The original HAADF-STEM images and c, d) HAADF-STEM polarization vector images of the N0 and N12 ceramics along with [100]_c_ zone axis.

**Figure S9.** Atomic-resolution energy-dispersive X-ray spectroscopy (EDS) maps of the N12 ceramics along with [100]_c_ zone axis.

**Figure S10.** Calculated *P*-*E* hysteresis loops of the a) N0 and b) N12 ceramics at room temperature using phase-field simulation method.

**References:**

[1] X. Zhou, Z. Liu, B. Xu, "Influence of dislocations on domain walls in perovskite ferroelectrics: Phase-field simulation and driving force calculation," *International Journal of Solids and Structures*, 238 (2022).

[2] S. Wang, M. Yi, B.-X. Xu, "A phase-field model of relaxor ferroelectrics based on random field theory," *International Journal of Solids and Structures*, 83 (2016) 142-153.

[3] Y. Li, L. Cross, L. Chen, "A phenomenological thermodynamic potential for BaTiO_3_ single crystals," *Journal of Applied Physics*, 98 (2005).
